# A Manual on Medical Botany of North America

**Published:** 1885-05

**Authors:** 


A MANUAL OF THE MEDICAL BOTANY OF NORTH AMERICA. By Laurence Johnson, A. M., M. D. New York: William Wood & Co., 56 and 58 Lafayette Place. 1884.

In the first part of this work, giving the elements of botany, the life-history of plants from germination to reproduction is presented.

In the second part, describing the Medicinal Plants of North

America, a systematic arrangement of most of the medicinal species, both indigenous and naturalized, is given.

The author has summarized the opinions of the most reliable observers, and also recorded his own experience in the notes upon medical properties and uses.

The book is gotten up in handsome style, with a number of elegant colored plates, and reflects much credit upon the publishers.

